# Influence of the bone graft materials used for guided bone regeneration on subsequent peri-implant inflammation: an experimental ligature-induced peri-implantitis model in Beagle dogs

**DOI:** 10.1186/s40729-022-00403-9

**Published:** 2022-01-21

**Authors:** Ryo Sato, Takanori Matsuura, Tatsuya Akizuki, Shunsuke Fukuba, Munehiro Okada, Kohei Nohara, Shunsuke Takeuchi, Shu Hoshi, Wataru Ono, Kiichi Maruyama, Yuichi Izumi, Takanori Iwata

**Affiliations:** 1 Department of Periodontology, Graduate School of Medical and Dental Sciences, Tokyo Medical and Dental University, 1-5-45 Yushima, Bunkyo-ku, Tokyo, 113-8510 Japan; 2 Department of Dentistry and Oral-Maxillofacial Surgery, School of Medicine, Fujita Health University, Aichi, Japan; 3 Hoshi Dental Clinic, Niigata, Japan; 4 Department of Oral Diagnosis and General Dentistry, Graduate School of Medical and Dental Sciences, Tokyo Medical and Dental University, Tokyo, Japan; 5 Oral Care Perio Center, Southern Tohoku Research Institute for Neuroscience, Southern Tohoku General Hospital, Fukushima, Japan

**Keywords:** Guided bone regeneration, Peri-implantitis, Autograft, Deproteinized bovine bone mineral, Animal study

## Abstract

**Purpose:**

We aimed to histologically evaluate the influence of bone materials used during guided bone regeneration (GBR) on subsequent peri-implantitis in an experimental ligature-induced peri-implantitis model in beagle dogs.

**Methods:**

Bilateral mandibular premolars (PM2-4) were extracted from six beagle dogs. After 3 months, standardized bone defects (3 mm [mesio-distal width] × 2 mm [bucco-lingual width] × 3 mm [depth]) were created in the experimental group, with simultaneous dental implant placement at the center of the defects. The defects were randomly filled with either autograft (AG) or deproteinized bovine bone mineral (DBBM) and covered with a collagen membrane. In the control group, implant fixtures were placed without creating an intrabony defect. After 3 months, a healing abutment was placed. Four weeks later, a 3–0 silk thread was ligated around the implants to induce peri-implantitis. After 4 weeks, the specimens were dissected and histologically examined.

**Results:**

There were no clinical findings of inflammation until silk thread ligation. Four weeks after the onset of peri-implantitis, gingival redness and swelling were seen with mild resorption of the peri-implant bone on dental radiographs. There were no significant differences between the AG, DBBM, and control groups for the following parameters: bone-to-implant contact, distance from the implant shoulder to the base of the bone defect, area of bone defect, and area of new bone.

**Conclusions:**

Within the limitations of this study, it can be concluded that peri-implant tissues after GBR using AG and DBBM underwent the same degree of bone resorption by peri-implantitis as the no defect group.

## Background

Placement of dental implants in the ideal three-dimensional position is important to achieve predictable functional and esthetic restoration [1, 2]. Following tooth extraction as a pre-procedure to implant treatment, the volume of the residual bone decreases, as is represented by buccal bone loss, due to alveolar bone remodeling [3, 4]. Horizontal and vertical bone resorption of up to 29–63% and 11–22%, respectively, has been reported within 6 months after tooth extraction [5]. Bone loss at the implant site is a risk factor for bone resorption after implant placement [6, 7].

Various bone regenerative techniques are effective in improving implant survival rates and have been used to achieve long-term success [8, 9]. Guided bone regeneration (GBR) is an established technique for the horizontal and vertical augmentation of the ridge volume with long-term stability [10].

Bone graft materials, including autograft (AG), deproteinized bovine bone mineral (DBBM), allograft, and alloplast, have been applied in GBR [11]. Among them, only AG possesses all three properties of osteogenesis, osteoinduction, and osteoconduction, and is therefore considered the gold standard [7, 12, 13]. However, the use of other bone grafts is increasing in clinical practice due to the problems associated with autografts, including greater invasiveness of the procedure to obtain the graft from the donor site and the limited quantity of the graft that can be harvested [12]. The effectiveness of DBBM for vertical and horizontal bone augmentation due to its osteoconductive property, has been reported. However, it is not osteoinductive [14] and resorbs poorly in tissues, with an unknown rate of resorption [15].

Peri-implantitis is one of the complications of implant therapy. According to the 2008 consensus report by the 6th European Workshop on Periodontology, 28–56% of patients treated with implants develop peri-implantitis in 1 year of implant placement [16]. In 2017, Ogata et al. reported the development of peri-implantitis in 9.7% of 267 Japanese patients 3 years after implant placement [17].

Therefore, prevention of peri-implantitis is important, and plaque control is an effective method to reduce its incidence [18]. However, since there are concerns that reduced plaque control is a result of decreased salivary volume and function associated with physical conditions, such as aging [19, 20], the risk of peri-implantitis may be considered unavoidable. In addition, this risk persists even after treatment of the peri-implant tissues by GBR.

Although the use of various bone grafts has been reported for the treatment of peri-implantitis, it remains unclear whether the bone graft material used for ridge augmentation influences the development of subsequent peri-implantitis.

Kim et al. demonstrated the usefulness of a three-walled GBR experimental model in dogs as a reproducible model for periodontal regeneration [21], and the ligature-induced peri-implantitis model became the standard model for the study of the treatment and pathogenesis of peri-implantitis following the research by Schwarz et al. [22, 23, 24]. Nevertheless, an experimental model to study the influence of the bone graft on subsequently occurring peri-implant inflammation has not been established. Therefore, we adapted the established experimental models for this study. We performed histological examinations to determine if the bone graft material used for augmentation altered the progression and extent of subsequent peri-implant inflammation.

## Methods

**Fig. 1:**
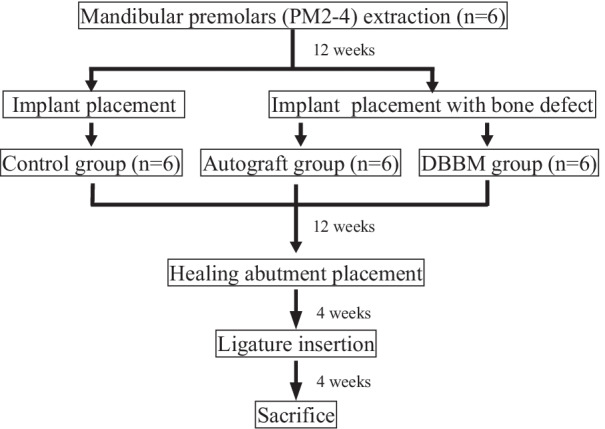
Diagram of experimental design protocol

All procedures and protocols in this study were approved by the Institutional Animal Care and Use Committee of Tokyo Medical and Dental University (A2018-322A) (Fig. 1). Six healthy 1-year-old male beagle dogs were used in this experiment. All surgical procedures were performed under general and local anesthesia. Medetomidine hydrochloride (0.05 mL/kg, Domitor®; Orion Corporation, Espoo, Finland) was administered intramuscularly as premedication. Spontaneous respiration was maintained by intravenous injection of sodium thiopental (0.005 mL/kg, Ravonal®; Mitsubishi Tanabe Seiyaku Co., Osaka, Japan). Lidocaine hydrochloride (2%, 1:80,000 epinephrine, Xylocaine; Fujisawa Pharmaceutical Co., Osaka, Japan) was administered as local anesthesia.

**Fig. 2:**
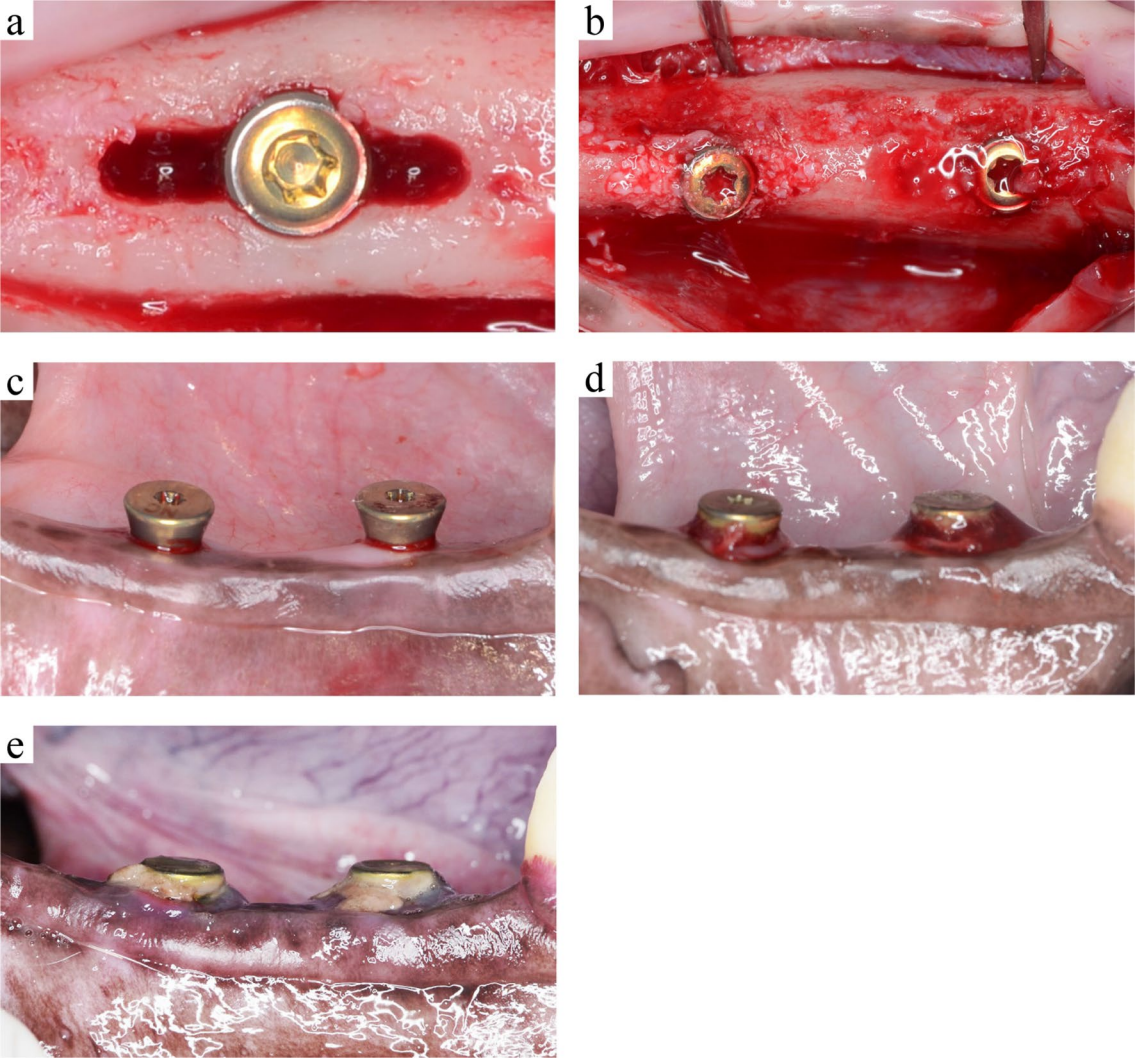
Surgical procedures. **a** Intrabony defects (3 mm [mesio-distal width] × 2 mm [bucco-lingual width] × 3 mm [depth]) were created and implants were placed at their center. **b** Left-sided defects were filled with deproteinized bovine bone minerals. Right-sided defects were filled with autograft. **c**Twelve weeks after implant placement, the cover screw was removed and the healing abutment was placed. **d** Four weeks after placing the healing abutment, a 3–0 silk ligature was placed. **e** Four weeks after placing the ligature to induce peri-implantitis, plaque accumulation and gingival redness were observed

Bilateral mandibular second, third, and fourth premolars in the beagles were extracted to provide sufficient space for dental implant placement. After 12 weeks of spontaneous healing, mucoperiosteal flaps extending from the first premolar to the first molar were elevated along the crest of the alveolar ridge. The alveolar crest was flattened by a periodontal chisel (Jovanovic; Hu-Friedy, Chicago, IL, USA) to obtain sufficient bone width for the experiment. The bone harvested during this process was collected for use as an AG. In the experimental group, standardized bone defects (2 mm buccal-lingual width × 3 mm mesio-distal width × 3 mm depth) were surgically created on the planned implant site. After the creation of these defects, bone-level implant fixtures (Straumann φ3.3 mmNC, SLA®8 mm, Roxolid®) were placed at the center of the defects (Fig. 2a). The intrabony defects were filled with either AG or DBBM (Bio-Oss®; Geistlich Pharma., Switzerland) (Fig. 2b). In the control group, implant fixtures were placed without creating an intrabony defect first. The experimental groups and the control group were designated randomly by random function (Microsoft Excel 2011; Microsoft Corporation, Redmond, WA, USA). After placing the cover screw (Straumann NC Closure cap φ2.8 mm, H 0 mm), an absorbable collagen membrane (Bio-Gide®; Geistlich Pharma., Switzerland) was placed in the experimental groups. The flaps were repositioned and immobilized using sutures (Gore-Tex® CV-6 Suture; W.L. Gore & Associates, Inc., Newark, DE, USA). After the surgical procedures, an antibiotic (Penicillin G; Meiji Seika Pharma Co., Ltd, Tokyo, Japan) and an analgesic agent (Vetorphale; Meiji Seika Pharma Co., Ltd, Tokyo, Japan) were administered intramuscularly. After 2 weeks of healing, the sutures were removed. The surgical site was rinsed with a 2% solution of chlorhexidine (HiBiTane® concentrate; Sumitomo Seiyaku Co., Ltd., Osaka, Japan) three times a week for 12 weeks.

Twelve weeks after implant placement, the position of each implant fixture was confirmed by bone sounding, and the gingiva over the cover screw was removed. The cover screw was replaced by the healing abutment (Straumann NC conical shape, φ4.8 3.5 mm) (Figs. 2c).

After 4 weeks of healing, 3–0 silk ligatures (Blade silk; Hashimoto Co., Ltd., Tokyo, Japan) were placed in the peri-implant sulcus (Fig. 2d). The remaining ligature thread was checked, and dental radiographs were taken once a week to confirm bone resorption around the implant. Four weeks after ligation, the beagles were euthanized by an overdose of intravenous thiopental. The mandibules containing all surgical sites were dissected into blocks and fixed in 10% neutral buffered formaldehyde (Mild form® 10 N; Wako Pure Chemical industries) for morphological and histological evaluation. One experienced surgeon (T.M.) performed all surgical procedures in this experiment.

### Radiographic analysis

The height of the alveolar bone around the implant fixture at the time of ligature placement and 4 weeks after ligation was compared on radiographs. The difference in the height was defined as the length of bone loss. In all groups, the length of bone loss was represented the average of the mesial and distal measurements. The ratio of the length of bone loss to the length of the implant fixture was calculated.

### Histological analysis

The tissue blocks containing the implant were fixed in 10% formaldehyde solution (Mildform® 10 N; Wako Pure Chemical Industries, Ltd.), followed by dehydration with ethanol solutions of different concentrations, which were replaced with acetone. After further treatment with methylmethacrylate (MMA) solution, the specimens were embedded in MMA resin, polished into 30–40 μm thick non-demineralized sections, and stained with toluidine blue.

Histological examination was done using an optical microscope (ECLIPSE Ni-U; Nikon Corporation, Tokyo, Japan). Histological measurements were performed using a computerized imaging system consisting of a high-definition color camera head (DS-Fi2; Nikon Corporation, Tokyo, Japan). The following parameters were measured by the same experienced and blinded examiner (T.A.). Intra-examiner reproducibility was ensured by the examiner reading 18 sections from all sites and repeating the same procedure 24 h later. Calibration was accepted at 90% level.

Bone-to-implant contact (BIC) was calculated as the percentage of implant-bone contact within the region of interest (ROI). An ROI was defined as the same area of the bone defect (3 mm mesio-distal width × 3 mm depth). The percentage of new bone and bone defect areas in the ROI were calculated. All reported parameters represented the average of the mesial and distal measurements. These items were measured using image analysis software (ImageJ v.1.43u; National Institutes of Health, Bethesda, MD, USA).

The following parameters were evaluated for all specimens:

- BIC (%): bone contact percentage of the implant body length in the ROI
- First BIC (fBIC, mm): distance from IS to first bone-to-implant contact
- Area of bone loss (mm^2^): the area from the implant shoulder (IS) to the base of the defect
- Area of new bone (mm^2^): amount of new bone formation calculated from the base of the defect.

### Statistical analysis

Means and standard deviations for each parameter were calculated for each group, and statistical analysis for each group was performed using analysis of variance (Microsoft Excel 2011; Microsoft Corporation, Redmond, WA, USA). The level of significance was set at P < 0.05. The data are expressed as mean ± SD.

Difference in the area of new bone between the AG and DBBM groups was assessed with the paired t-test, and a p-value < 0.05 was considered statistically significant.

## Results

### Clinical observations

No flap necrosis or wound dehiscence was observed during the 12 weeks after implant placement. The residual bone grafts were not exposed at any of the experimental sites. Healing occurred uneventfully at all sites in all groups. All implants achieved good primary stability at the time of installation. There was no loss of implants during the experiment. Four weeks after ligation, plaque deposition and gingival redness were observed around the implants (Fig. 2e).

### Radiographic observations

**Fig. 3:**
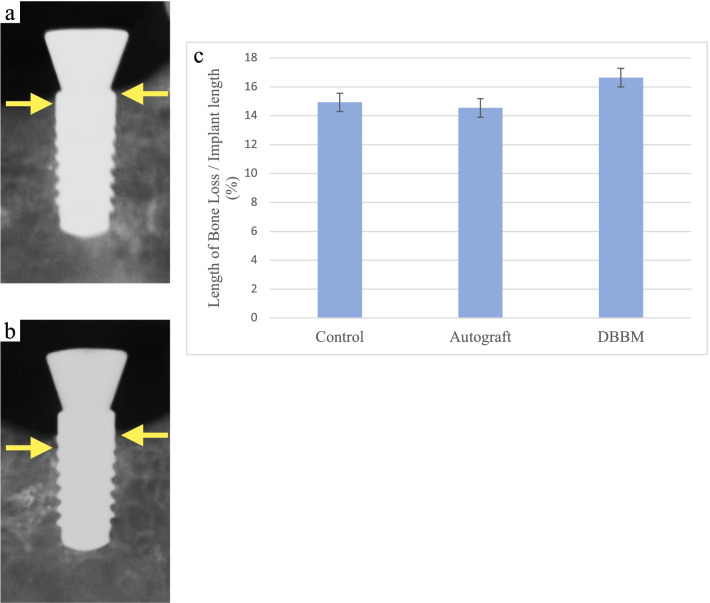
Dental radiographs and graphics representing the average bone loss relative to the implants on the radiographs. **a** Four weeks after placing the healing abutment. Yellow arrows represent the bottom of bone loss. **b** Four weeks after insertion of the ligature. Yellow arrows represent the bottom of bone loss. **c** Graphic about the average bone loss in all groups. There was no statistically significant difference used by the analysis of variance

At the time of insertion of the 3–0 silk thread, the heights of the alveolar bone and IS were comparable in all groups (Fig. 3a). There was no continuous radiolucency around the implants. No bone graft was identified in the AG and DBBM groups. Four weeks later, the bone level of the alveolar ridge in all groups was below IS (Fig. 3b). The average bone loss relative to the implants was 14.3 ± 0.9% in the control group, 13.6 ± 1.9% in the AG group, and 14.8 ± 2.1% in the DBBM group, and there was no statistically significant difference (Fig. 3c).

### Histological observations

**Fig. 4:**
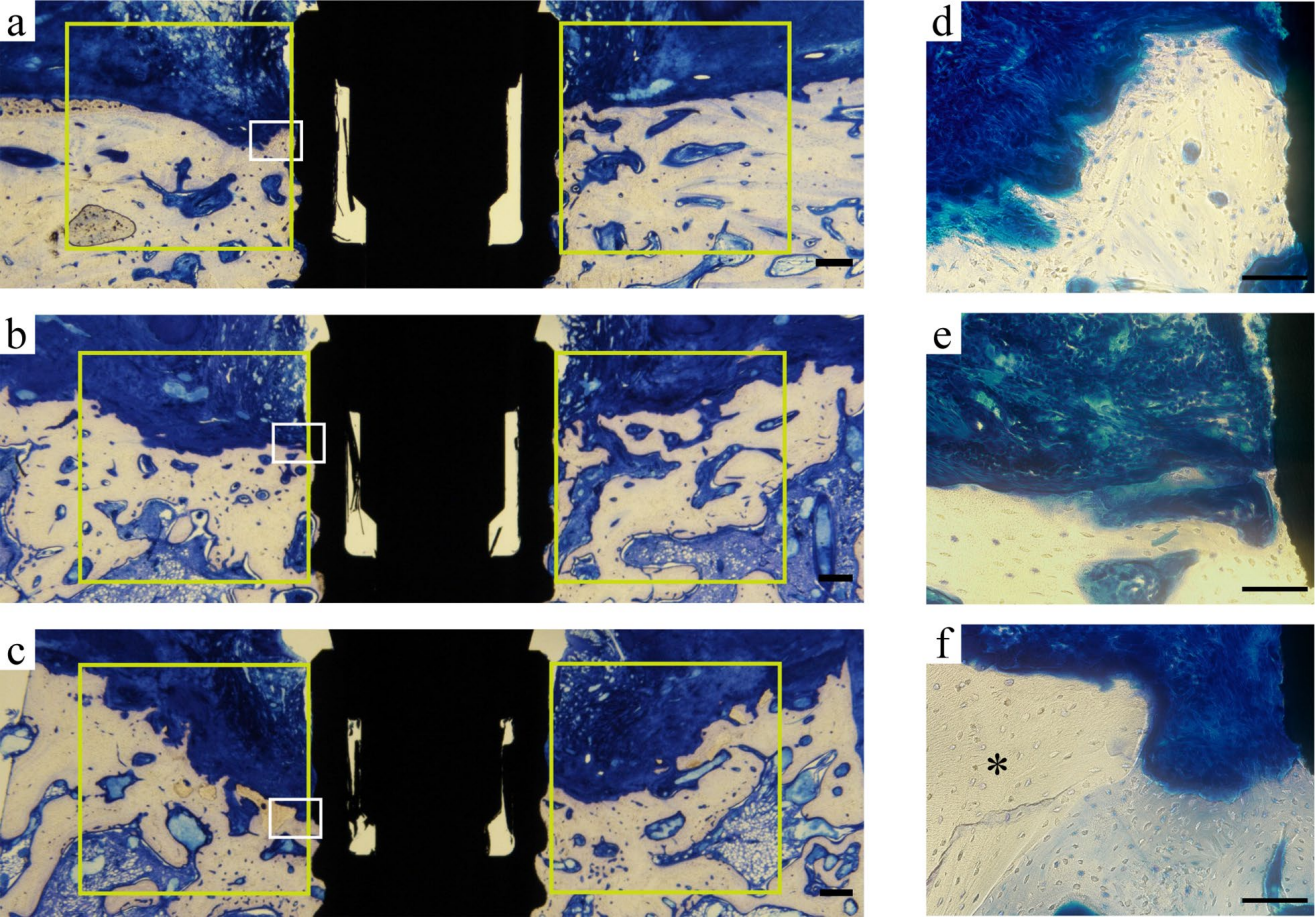
Photomicrograph of a region of interest (Toluidine blue). **a**–**c** Original magnification view (Scale bar = 500 μm). The area inside the yellow box indicates the region of interest. **d**–**f** The white boxed depicts the tissue under high magnification (× 20, Scale bar = 50 μm). **a**, **d** Control, **b**, **e** autograft, and **c**, **f** deproteinized bovine bone mineral groups. *Residual bone graft

Shallow circumferential bone loss around implants was observed in all groups. No fibrous tissue intervention was found around the implants (Fig. 4).

Compared to the control group, the bone distribution around the implants in the AG group was sparse. The boundary between new bone, AG, and original bone was unclear. (Fig. 4a, b).

In the AG group, residual bone graft was not observed. The AG had been replaced by alveolar bone (Fig. 4b, e). Residual bone grafts of DBBM were found within the ROI and encased in bone tissue (Fig. 4c, f).

### Histometric analyses

No significant differences in bone defect area, new bone area, BIC, and fBIC were observed between the control, AG, and DBBM groups (Table 1). Although there was no statistically significant difference, there was a tendency for the DBBM group to have a lower BIC and a higher fBIC.

**Table 1:** Results of morphometric measurements of BIC, fBIC, and area of bone loss

	Control group	AG group	DBBM group
BIC (%)	60.1 ± 1.92	61.2 ± 3.41	55.6 ± 4.51
fBIC (mm)	1.19 ± 0.07	1.16 ± 0.10	1.33 ± 0.16
Area of bone loss (mm^2^)	2.80 ± 0.43	3.21 ± 0.31	3.52 ± 0.21

One-way ANOVA was performed. Statistical significance was determined at p < 0.05. There were no statistically significant differences between groups for all parameters

*BIC* bone-to-implant contact, *fBIC* first BIC, *AG* autograft, *DBBM* deproteinized bovine bone mineral. Average ± SD. n = 6

The area of bone loss was greater in the AG and DBBM groups than in the control group.

The area of new bone area was greater in the AG group than in the DBBM group. But there was no statistically significant difference (P = 0.056) (Table 2).

**Table 2:** Results of morphometric measurements of area of new bone formation

	AG group	DBBM group
Area of new bone (mm^2^)	5.79 ± 0.31	5.14 ± 0.21

The paired t-test was performed. There was no statistically significant difference

*AG* autograft, *DBBM* deproteinized bovine bone mineral. Average ± SD. p < 0.05; n = 6

## Discussion

This study histologically investigated the influence of different bone grafts (AG and DBBM) on peri-implant inflammation after GBR. Schwarz et al. demonstrated that peri-implantitis progresses faster than periodontitis because of the structural differences between the periodontal and peri-implant tissues [25]. Hämmerle et al. reported that the survival rate of implants placed at grafted sites was similar to that of implants not requiring guided bone regeneration [26]. However, it is unclear if the graft used for ridge augmentation with GBR can influence subsequent peri-implant inflammation.

To our knowledge, this is the first study to histologically evaluate the inflammation in peri-implant tissues regenerated by GBR; therefore, we combined the previous GBR model with the experimental peri-implantitis model.

This study compared the effect of inflammation on the AG and DBBM grafted concomitantly as implant placement in comparison with that in the control group. Therefore, no bone defect was created in the control group. The size of the bone defect to be fabricated was small because the bone height needed to be comparable in all groups before induction of peri-implantitis. In fact, the dental radiographs acquired before peri-implantitis induction showed that the bone levels were similar in height in all the groups.

In previous studies, active breakdown of ligature-induced peri-implantitis has been reported over a mean period of 12.0 ± 5.0 weeks, with a mean bone loss of 41.6 ± 16.1% relative to the implant length [22, 27]. Compared to these previous reports, the ligation period in the present study was shorter and the amount of bone resorption observed was lesser. In this study, a bone resorption of 40% of the 8-mm-long implant could result in a greater extent of resorption than that with the original 3-mm-deep bone defect. As a result, it could be difficult to assess the effect of inflammation-induced bone resorption on the area where the bone substitute was implanted. Therefore, in this study, the ligation period was scheduled to be shorter than that in previous studies to ensure that the extent of bone resorption would not exceed the implanted bone substitute [22, 27].

Some studies reported that DBBM particles can induce the expression of multinucleated giant cells, or stimulate the formation of fibrous tissue [28–30]. In other studies, DBBM particles were not absorbed under inflammatory conditions [28, 31]. In the present study, residual DBBM granules were observed in the DBBM group, confirming the insolubility of DBBM, as reported by Handschel et al. [15] (Fig. 4). Multinucleate giant cells were not observed around the DBBM granules in the histological sections.

Encapsulation by fiber tissue occurs in response to foreign bodies [29]. If the graft is not encapsulated in soft tissue, GBR is considered successful [29, 32]. In this study, since encapsulation was not observed, we considered GBR to be successful. After DBBM was surrounded by new bone, the progress of bone resorption was same as that of the native bone.

Comparing the BIC in the control and AG groups, there was lower BIC in the DBBM group. However, the difference was not statistically significant. Santis et al. reported that BIC tended to be lower in the DBBM group than the AG group, but there was no significant difference [33]. Other studies reported that bone resorption in areas augmented by AG was unpredictable and could vary between 12 and 80% [34]. Since DBBM particles are stable and induce bone formation in the long term, DBBM may be more advantageous for the long-term prognosis [35]. It was suggested that the use of DBBM in combination with AG may improve the stability of the graft [34]. However, it has been reported that bone at the bone-implant interface has weak mechanical properties due to its low mineral content [36]. Compared to the control and AG groups, the DBBM group tended to have a larger area of bone loss and fBIC, which could be influenced by the mineral content. GBR using AG or DBBM may be an effective treatment in terms of resistance of peri-implantitis.

The DBBM group showed a tendency to show lower results for all parameters compared to the other groups; however, the difference was not significant. It is thought that the residual granules of DBBM did not influence the degree of bone resorption due to inflammation as a sufficient healing period was provided after GBR. This suggests that the long-term prognosis of inflammation is not dependent on the bone graft used for GBR. In this study, peri-implantitis was not induced immediately after GBR, so it is unclear whether inflammation generated during bone regeneration affects DBBM. To investigate the influence of inflammation on bone graft materials, further research is needed to determine the short-term prognosis.

Further investigation of the inflammation in peri-implant tissues after GBR using different bone grafts, such as allografts and alloplasts, is needed.

In this study, there was no difference in the bone resorption by the inflammation occurring in the AG and DBBM groups compared to control group, indicating the usefulness of DBBM with regard to good availability of the graft without requiring invasive procedures.

## Conclusion

Within the limitations of this study, it can be concluded that peri-implant tissues after GBR using AG and DBBM underwent the same degree of bone resorption by peri-implantitis as the no defect group.

## Data Availability

All data generated or analyzed during the current study are available from the corresponding author upon reasonable request.

## Abbreviations

AG: Autograft
DBBM: Deproteinized bovine bone mineral
BIC: Bone-to-implant contact
fBIC: First BIC
GBR: Guided bone regeneration
ROI: Region of interest
